# Association between hair cortisol concentration and metabolic syndrome

**DOI:** 10.1515/med-2021-0298

**Published:** 2021-06-16

**Authors:** Eglė Mazgelytė, Asta Mažeikienė, Neringa Burokienė, Rėda Matuzevičienė, Aušra Linkevičiūtė, Zita Aušrelė Kučinskienė, Dovilė Karčiauskaitė

**Affiliations:** Department of Physiology, Biochemistry, Microbiology and Laboratory Medicine, Institute of Biomedical Sciences, Faculty of Medicine, Vilnius University, Vilnius, Lithuania; Clinics of Internal Diseases, Family Medicine and Oncology, Institute of Clinical Medicine, Faculty of Medicine, Vilnius University, Vilnius, Lithuania

**Keywords:** cortisol, metabolic syndrome, psychosocial stress

## Abstract

Metabolic syndrome (MetS) is a highly prevalent disorder defined as a cluster of cardiometabolic risk factors including obesity, hyperglycemia, hypertension, and dyslipidemia. It is believed that excessive cortisol secretion due to psychosocial stress-induced hypothalamic-pituitary-adrenal axis activation might be involved in the pathogenesis of MetS. We sought to explore the association between MetS and psychosocial risk factors, as well as cortisol concentration measured in different biological specimens including saliva, blood serum, and hair samples. The study was conducted on a sample of 163 young and middle-aged men who were divided into groups according to the presence of MetS. Hair cortisol concentration (HCC) was determined using high performance liquid chromatography with UV detection, while blood serum and salivary cortisol levels were measured by enzyme-linked immunoassay. Lipid metabolism biomarkers were determined using routine laboratory methods. Anthropometric and lifestyle characteristics, as well as self-reported psychosocial indicators, were also examined. Significantly higher HCC and lower social support level among participants with MetS compared with individuals without MetS were found. However, no significant differences in blood serum and salivary cortisol levels were observed between men with and without MetS. In conclusion, chronically elevated cortisol concentration might be a potential contributing factor to the development of MetS.

## 1 Introduction

Metabolic syndrome (MetS) is a cluster of metabolic abnormalities including abdominal obesity, hyperglycemia, hypertension, reduced high-density lipoprotein cholesterol (HDL-C), and elevated triacylglycerol (TAG) concentration [1]. MetS is associated with a 5-fold increased risk for type 2 diabetes and two times higher risk for the development of cardiovascular diseases which are the leading cause of death worldwide [1,2]. It is estimated that about one quarter of the world population is affected with MetS. The cost of MetS including informal care provided by family and direct costs of medical care, as well as loss of potential economic activity, is in trillions [3]. Moreover, MetS has become increasingly prevalent among young and middle-aged adults living in economically developed countries [4]. Although the pathogenesis of MetS is not fully elucidated, it is likely that there is an interaction between metabolic, genetic, and environmental factors [5].

There is some evidence suggesting that long-term and intense stress or experience of extremely stressful life events (e.g., disaster) is associated with the elevated risk of MetS onset [6,7]. Stress-induced activation of hypothalamic-pituitary-adrenal (HPA) system results in the production of cortisol, the main glucocorticoid in humans. Since chronically elevated cortisol concentration promotes abdominal obesity, hypertension, and hyperglycemia, it is believed that stress should be considered as an etiological factor of MetS [8,9,10]. However, the existing literature on the relationship between cortisol concentration and MetS is inconsistent. Some studies found positive association of cortisol concentration with the prevalence of MetS [11,12], while other papers reported no association [13,14] or even negative relationship between cortisol concentration and MetS [15]. Similarly, distinct findings on the association between stress-related psychosocial factors such as social support or work stress and MetS have been observed. Several studies have found significant association of lower social support level and higher work-related stress with the increased prevalence of MetS [16,17], while other large studies demonstrated no relationship [18] or gender-specific associations between these psychosocial indicators and MetS [19]. In the previous meta-analysis, significant associations between higher perceived stress level and the prevalence of individual MetS parameters (i.e., visceral obesity, dyslipidemia, hypertension) were found. Interestingly, no relationship between the perceived stress and the presence of MetS diagnosis was detected [20]. Another systematic review and meta-analysis revealed the importance of the stress source and found that the strongest impact on the MetS risk is attributed to occupational stress, while general stress or stressful life events were not related to the increased prevalence of MetS [21].

The most common approach for the objective evaluation of stress level is measurement of cortisol concentration in blood serum or saliva samples [22]. The collection of saliva specimens is easily performed, noninvasive, painless, and relatively stress-free, while blood collection requires qualified medical personnel and venipuncture-induced stress might give falsely higher cortisol concentrations [23]. In addition, salivary cortisol concentration reflects the circulating level of free, biologically active fraction of hormone rather than total levels, which are confounded by the presence of high affinity binding proteins [24,25]. Nevertheless, both salivary and blood serum cortisol concentrations indicate acute or short-term changes in HPA axis activity. In the last decade, the analysis of cortisol in human scalp hair has received an increasing attention as a promising chronic stress biomarker since it represents long-term (1–3 months) HPA axis activity [23,24,26,27,28]. We hypothesize that the presence of conflicting results on the relationship between HPA axis activity and MetS might be caused by different biological matrices (i.e., saliva, blood serum or plasma, hair) used for the evaluation of cortisol concentration. Thus, the major objective of this study was to explore the associations between MetS and cortisol concentration measured in different biospecimens including blood serum, saliva, and hair samples in young and middle-aged men. Also, we aimed to analyze differences in subjectively evaluated psychosocial factors between men with and without MetS.

## 2 Methods

### 2.1 Study population and procedure

This cross-sectional study included 163 young and middle-aged (25–55 years) men, who were recruited consecutively from the database of the Outpatient Department of Vilnius University Hospital Santaros Klinikos. Individuals with mental and endocrine disorders were not involved in the study. Also, subjects were excluded if they used synthetic glucocorticoids during the previous 3 months. Data collection was implemented by appropriately trained general practitioner and nurses working at the Outpatient Department of Vilnius University Hospital Santaros Klinikos, a public tertiary healthcare institution in Lithuania. During the first visit in the healthcare institution, each enrolled individual filled out the psychosocial stress questionnaire validated in the LiVicordia study [29], as well as a questionnaire on sociodemographic and lifestyle characteristics, including age, education level, monthly income, smoking status, physical activity, the presence of night shift work, and additional job. Also, Salivette^®^ devices with the manufacturers’ instructions were given for each study participant and subjects were asked to obtain their saliva samples immediately after awakening on the day of the second visit to the healthcare institution. The second data collection stage was scheduled in the morning (at 8:00–9:00 h) within a week after the first stage. On the second visit, subjects delivered their saliva samples. Also, blood samples for biochemical analysis, hair samples for cortisol concentration measurement, as well as anthropometric data were obtained by trained personnel. All participants provided written informed consent, and this research followed the tenets of the Declaration of Helsinki published in 1964 and its later amendments and also the study protocol was approved by the Lithuanian Bioethics Committee (No. 15820-15-807-319).

### 2.2 Psychosocial stress questionnaire

The questionnaire consisted of four major parts including job strain, social support, personality, and depression. Job strain was evaluated as a combined effect of psychological demands at work and authority over decisions (demand/control). Social support score consisted of questions about social support at the work site and global social support with the two dimensions, emotional support and social integration. Personality score was calculated using instruments on coping, self-esteem, sense of coherence, hostility, immersion, and vital exhaustion ((coping + self-esteem + sense of coherence)/(hostility + immersion + vital exhaustion)). Depression was estimated using 13-item Beck depression inventory [30].

### 2.3 Biochemical analyses and MetS diagnosis

All blood samples were collected under fasting conditions and were analyzed in the Centre of Laboratory Medicine of Vilnius University Hospital Santaros Klinikos. Specifically, HDL-C, TAG, and glucose concentration in blood serum were determined using routine laboratory methods (Architect ci8200, Abbott, USA). Anthropometric assessment involved waist circumference (WC) and resting arterial blood pressure (systolic and diastolic) measures. MetS diagnosis was based according to the International Diabetes Federation consensus worldwide definition of the MetS [31]. MetS was diagnosed if an individual had central obesity (WC ≥ 94 cm) and any two of the following four factors: raised TAG concentration (≥1.7 mmol/L), reduced HDL-C concentration (<1.03 mmol/L) or specific treatment for these lipid abnormalities, increased arterial blood pressure (systolic BP ≥ 130 or diastolic BP ≥ 85 mm Hg) or treatment of previously diagnosed hypertension, elevated fasting plasma glucose concentration (≥5.6 mmol/L), or previously diagnosed type 2 diabetes. Arterial hypertension was diagnosed according to the guidelines of the International Society of Hypertension [32].

### 2.4 Analysis of stress biomarkers

#### 2.4.1 Determination of cortisol concentration in saliva and blood serum samples

Saliva samples were collected using Salivette^®^ devices (Sarstedt Co. Ltd., Rommelsdorf, Germany). Saliva samples were stored at −80°C. After thawing, samples were centrifuged for 10 min at 4,000 rpm. Fasting blood samples for cortisol measurement were collected into vacuum tubes (BD Vacutainer SST II Advance (Becton Dickinson, USA)) in the morning (between 7 and 8 am). After collection, blood samples were centrifuged for 10 min at 3,000 rpm. Blood serum samples were stored at −80°C until analysis. Cortisol concentration in blood serum and saliva samples were determined using commercial ELISA kits (LDN^®^, Nordhorn, Germany). The sensitivity of ELISA assay for the quantitative determination of cortisol in blood serum was 1.3 ng/mL, while the sensitivity of ELISA for the cortisol measurement in saliva was 0.019 ng/mL.

#### 2.4.2 Determination of cortisol concentration in human hair

Hair cortisol concentration (HCC) was determined from the most proximal segment of 3 cm of scalp hair, representing approximately 3 months prior to sampling grown hair. The hair samples were stored at room temperature in envelopes until analysis. Samples were prepared using slightly modified methods published by Raul et al. [33] and de Palo et al. [34]. Hair samples were washed twice in 5 mL isopropanol. A 20–50 mg of each sample was finely cut with scissors into small fragments (∼1 mm long) to improve the efficiency of extraction and incubated in 1.5 mL of Sorenson’s buffer, pH 7.6, for 16 h at 40°C, in the presence of 10 ng of 6-α methylprednisolone as internal standard. Each sample then was transferred to solid-phase extraction Discovery DSC-18 column (Sigma-Aldrich, St. Louis, USA), which was previously equilibrated (3 mL MeOH followed by 1.5 mL of water). The subsequent steps were the following: washing with 0.5 mL of water followed by 0.5 mL of acetone/water (1:4, v/v), 0.25 mL of hexane, and elution with 1.5 mL of diethyl ether. The eluates were evaporated under a stream of nitrogen gas and resuspended with 100 μL of acetonitrile/water (1:1, v/v). Cortisol concentration was determined using Shimadzu Nexera X2 UHPLC system (Shimadzu Corp., Kyoto, Japan). A 10 μL of the extract was injected on the Zorbax Eclipse XDGB-C8 (5.0 μm, 4.6 × 150 mm; Agilent Technologies) column. The chromatographic isocratic separation was carried out with a binary mobile phase of acetonitrile and deionized water (2:3, v/v). The flow rate was 1.0 mL/min. The UV absorbance was measured at 245 nm wavelength. The average retention time of the cortisol was 4.12 min. Data were collected and processed using the LabSolutions software (Shimadzu Corp.).

## 3 Statistical analysis

Statistical analysis was performed with R version 3.6.0. Quantitative variables are presented as median (interquartile range) (IQR), while absolute and relative frequencies were calculated for categorical variables. Chi-square test was employed to compare the categorical variables between men with and without MetS, as well as to analyze the differences of MetS prevalence among study participants stratified into groups based on their HCC and social support level. The strength of association between categorical variables was evaluated by calculating contingency coefficient (*C*). Furthermore, Mann–Whitney *U* test was used for the comparison of continuous variables. Spearman’s rank coefficient was used to quantify the strength of the correlation between HCC and criteria of MetS. Binary simple and multivariable logistic regression analyses were performed to evaluate predictors of MetS. The level of statistical significance was set at 0.05 for two-tailed testing.

## 4 Results

### 4.1 Sample characteristics

Table 1 shows the descriptive characteristics of the study sample. Thirty eight (23.3%) of participants met the criteria of MetS. MetS patients were significantly older and less physically active during leisure time compared with participants without MetS. In contrast, there were no significant differences regarding education level, income, smoking status, physical activity at work, and the prevalence of night shift work or additional job between MetS patients and healthy men. The comparison of psychosocial stress indicators showed significantly lower social support level in MetS patients than in the group of participants without MetS. Regarding the objective psychosocial stress measures, only HCC median values differed significantly among MetS patients and healthy individuals.

**Table 1:** Comparison of sociodemographic, lifestyle, psychosocial indicators, and stress biomarkers between individuals with and without MetS

Characteristics	Individuals without MetS (*n* = 125)	MetS patients (*n* = 38)	*χ* ^2^, df = 1	*p*-value
**Sociodemographic and lifestyle indicators**				
Age (years), median (IQR)	35 (18)	42.5 (10)		**0.007**
Education level (university graduates or those with higher education), *n* (%)	119 (95.2)	33 (91.7)	0.66	0.416
Income (higher than national average monthly wage), *n* (%)	104 (83.2)	32 (88.9)	0.69	0.406
Smoking status (current smoker), *n* (%)	18 (14.5)	9 (25.0)	2.19	0.139
Physical activity at work (physically active), *n* (%)	35 (28.0)	14 (38.9)	1.57	0.211
Recreational physical activity (physically active), *n* (%)	109 (87.2)	22 (62.9)	10.69	**9.520 × 10** ^**−4**^
Additional job, *n* (%)	28 (22.4)	11 (30.6)	1.01	0.314
Night shift work, *n* (%)	18 (15.5)	2 (4.5)	2.05	0.250
**Psychosocial indicators**				
Depression, median (IQR)	2.00 (5)	3.00 (4)		0.804
Personality, median (IQR)	0.51 (0.10)	0.52 (0.10)		0.901
Job strain, median (IQR)	0.67 (0.21)	0.72 (0.23)		0.384
Social support, median (IQR)	48.00 (10.0)	46.50 (9.25)		**0.009**
**Stress biomarkers**				
Hair cortisol concentration (ng/g), median (IQR)	36.50 (98.26)	85.73 (150.88)		**0.005**
Morning salivary cortisol concentration (ng/mL), median (IQR)	9.16 (6.78)	11.09 (9.85)		0.193
Cortisol concentration in blood serum (ng/mL), median (IQR)	221.78 (94.29)	200.62 (128.15)		0.168

Note: Statistically significant *p*-values (<0.05) are shown in bold font.

### 4.2 HCC, social support level, and MetS

Table 2 represents the correlation between HCC and distinct criteria of MetS. We found significant relationship between HCC and participants’ WC, resting systolic and diastolic blood pressure values, and fasting glucose concentration. However, there was no evidence for correlations between HCC and HDL-C or TAG concentration in serum samples. Correlation analysis also showed significant associations between subjectively perceived social support level and WC values, as well as fasting glucose concentration in blood serum (Table 3).

**Table 2:** Correlations between HCC and criteria of metabolic syndrome

Variable	Spearman’s *r*	*p*-value
Waist circumference (cm)	0.21	**0.007**
Resting systolic blood pressure (mm Hg)	0.34	**9.55 × 10** ^**−6**^
Resting diastolic blood pressure (mm Hg)	0.32	**3.05 × 10** ^**−5**^
Fasting glucose (mmol/L)	0.16	**0.046**
High-density lipoprotein cholesterol (mmol/L)	−0.03	0.746
Triacylglycerols (mmol/L)	0.11	0.144

Note: Statistically significant *p*-values (<0.05) are shown in bold font.

**Table 3:** Correlations between subjectively perceived social support level and criteria of metabolic syndrome

Variable	Spearman’s *r*	*p*-value
Waist circumference (cm)	−0.14	**0.044**
Resting systolic blood pressure (mm Hg)	−0.03	0.629
Resting diastolic blood pressure (mm Hg)	−0.12	0.065
Fasting glucose (mmol/L)	−0.14	**0.040**
High-density lipoprotein cholesterol (mmol/L)	0.10	0.139
Triacylglycerols (mmol/L)	−0.04	0.557

Note: Statistically significant *p*-values (<0.05) are shown in bold font.

**Figure 1:**
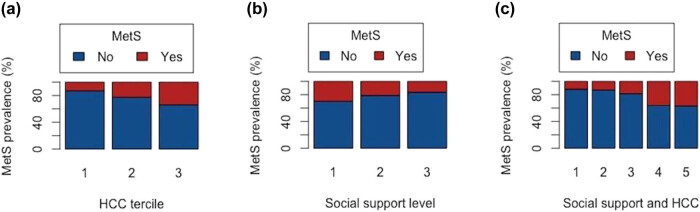
The prevalence of metabolic syndrome (%) according to HCC (a), social support level (b) terciles and five groups based on both HCC tercile and social support level (c).

Since significant differences in HCC and social support levels were found between healthy individuals and MetS patients, we divided the entire study sample into three groups according to HCC and social support level terciles. Specifically, 1st, 2nd, and 3rd HCC tercile indicates low, moderate, and high chronic stress level, respectively. Similarly, 1st, 2nd, and 3rd social support tercile means low, moderate, and high subjectively perceived social support level, respectively. The prevalence of MetS (%) significantly increased with HCC, expanding from 13.0 to 33.9% from the first to the third terciles (*χ* ^2^ = 6.78, *p* = 0.034) (Figure 1a). Furthermore, we found statistically significant association between HCC terciles and the prevalence of MetS (contingency coefficient *C =* 0.200, *p* = 0.033). Although the frequency of MetS diagnosis decreased from the 1st to the 3rd social support level tercile (from 29.8 to 16.3%) (Figure 1b), the *χ* ^2^ test of independence showed that MetS diagnosis was independent of social support level (*χ* ^2^ = 2.85, *p* = 0.241) and its contingency coefficient value was nonsignificant (*C* = 0.133, *p* = 0.241). To investigate the relationship between the prevalence of MetS and cumulative effect of chronic stress and social support level, we stratified participants into five groups (1st – low chronic stress and high social support level; 2nd – low chronic stress and moderate social support level or moderate chronic stress and high social support level; 3rd – moderate chronic stress and social support level; 4th – high chronic stress and moderate social support level or moderate chronic stress and low social support level; 5th – high chronic stress and low social support level). Results showed increase in MetS prevalence as going from the 1st to the 5th group (from 11.8 to 36.8%) (Figure 1c) (*χ* ^2^ = 9.18, *p* = 0.066) and contingency coefficient value barely below the level of significance (*C* = 0.235, *p* = 0.057).

We analyzed four logistic regression models: unadjusted (Model 1), age-adjusted (Model 2), age and recreational physical activity-adjusted (Model 3), and age, recreational physical activity, and social support level-adjusted (Model 4) models. The highest HCC tercile was associated with MetS in unadjusted (Model 1) and age-adjusted (Model 2) models. However, after adjustment for age (Model 2), the primary odds ratio found in unadjusted model (Model 1) fell to 2.77 (95% CI: 1.03, 7.49), while an additional adjustment for recreational physical activity (Model 3) resulted in the decrease of odds ratio to 2.60 which was close to being statistically significant. Furthermore, an adjustment for social support level has not changed the results significantly (Model 4). The prevalence of MetS was not significantly different among persons in the second HCC tercile compared with those in the lowest tercile in both unadjusted (Model 1) and adjusted models (Models 2–4) (Table 4).

**Table 4:** Logistic regression models predicting MetS prevalence based on HCC terciles

Hair cortisol terciles	MetS prevalence (%)	Model 1 OR (95% Cl) unadjusted	*p*-value	Model 2 OR (95% CI) adjusted for age	*p*-value	Model 3 OR (95% CI) adjusted for age and recreational physical activity	*p*-value	Model 4 OR (95% CI) adjusted for age, recreational physical activity, and social support	*p*-value
1	13.0	1.00 (referent)		1.00 (referent)		1.00 (referent)		1.00 (referent)	
2	22.6	1.97 (0.71, 5.46)	0.195	1.69 (0.59, 4.80)	0.325	1.31 (0.43, 3.96)	0.638	1.23 (0.40, 3.82)	0.716
3	33.9	**3.45 (1.31, 9.07)**	**0.012**	**2.77 (1.03, 7.49)**	**0.044**	2.60 (0.92, 7.40)	0.073	2.56 (0.90, 7.27)	0.078

Note: Statistically significant odd ratios (95% CI) and the corresponding *p*-values (<0.05) are shown in bold font.

## 5 Discussion

The main focus of this study was to evaluate the association of MetS prevalence with objective stress biomarkers and distinct psychosocial stress indicators in young and middle-aged men. Analysis of objective stress biomarkers revealed significantly higher HCC in MetS patients compared with participants without MetS. Also, stratification of HCC into terciles showed that higher HCC tercile is related to increased presence of MetS. These findings are in line with the previous research, showing that the prevalence of MetS was the highest in the third HCC tercile in the population of depressed patients and age- and gender-matched healthy individuals [12]. A recently published case-control study investigated the relationship between HCC and MetS, as well as PTSD and MetS co-occurrence in a population of South African mixed ancestry females. Authors reported no significant association of HCC with MetS or PTSD and MetS comorbidity [35]. These inconsistencies might arise from gender-specific effects, as well as other factors mediating the association between HCC and MetS. For instance, Lehrer et al. [21] found a direct negative association of psychological resilience and MetS severity. The more complex analysis using moderated mediation model indicated that indirect association between perceived stress and MetS via HCC varies as a function of psychological resilience [21]. Thus, factors potentially mediating the relationship between stress and MetS should be explored in the future studies.

We found significant correlations between HCC and distinct criteria of MetS including WC, arterial blood pressure, and fasting glucose concentration. These results support the idea that chronic glucocorticoid excess is manifested by increased adipogenesis of visceral fat, mineralocorticoid receptor-associated hypertension, and induced activities of gluconeogenic enzymes [36,37,38]. Similarly, Kuehl et al. [12] reported that HCC significantly correlated with WC, systolic blood pressure, and TAG concentration. Another study in large aerospace company employees showed significant positive associations of HCC with WC values and glycosylated hemoglobin level [11]. However, a cross-sectional study conducted among HIV-infected patients found no relationship between MetS and individual cardiometabolic measures, except the positive association of MetS with HDL-C concentration [15].

Since no differences in salivary and blood serum cortisol concentrations were observed between MetS patients and subjects without MetS under baseline conditions, our results are in accordance with the evidence that cortisol concentration measurements in hair, saliva, and blood serum samples serve as independent markers of HPA axis activity. Results from the previous research examining the associations between blood serum cortisol and MetS showed inconsistent results. For instance, Park et al. [39] found that increased MetS risk was associated with higher blood serum cortisol even after adjustment for age and body mass index in Korean adults. On the other hand, a study conducted in a sample of older Italian men demonstrated no significant relationship between MetS and cortisol concentration in blood serum [40]. In addition, a more recently published systematic review with meta-analysis of observational studies found no evidence of association between MetS and basal cortisol levels measured in saliva, blood serum, and urine samples [41]. The lack of such associations might be explained by the fact that cortisol concentration in biological fluids is dependent on tissue-specific cortisol metabolism including the rate of secretion, inactivation, and excretion [8]. For example, salivary glands possess the activity of 11β-hydroxysteroid dehydrogenase type 2 (11β-HSD2) enzyme which irreversibly converts cortisol to inactive cortisone [8,42,43]. Thus, diversity of 11β-HSD2 activity results in altered salivary cortisol concentration with cortisol to cortisone ratio ranging from 1:2 to 1:8 [43]. Moreover, it is suggested that increased tissue sensitivity to cortisol due to polymorphisms in glucocorticoid receptor (GR) gene (*NR3C1, Nuclear Receptor Subfamily 3 Group C Member 1*) is related to the criteria of MetS (e.g., visceral obesity, hypertension), despite normal HPA axis activity [8,41]. These observations emphasize that the link between HPA axis activity and MetS might be affected by variability in cortisol metabolism and tissue-specific sensitivity to glucocorticoids.

Our results showed that among psychosocial indicators, only subjectively perceived social support level differed significantly between MetS patients and participants without MetS. Specifically, significant negative correlations between social support level and WC, as well as fasting glucose concentration, were found. However, when social support level was treated as a categorical variable (i.e., low, moderate, high social support), no evidence of association with the prevalence of MetS was noticed. Similarly, Hwang and Lee [44] reported no significant relationship between the MetS diagnosis and social support level considered as a dichotomous variable in Korean male and female blue-collar workers. A previous study by Ortiz et al. [18] showed a lack of relationship between the prevalence of MetS and social support in U.S. Latino population. Few recently published studies also failed to show any evidence of association between MetS and social support level in a group of cancer caregivers and among medical university staff members [45,46]. In contrast, the SOPKARD study [17] on 476 citizens of Sopot demonstrated that frequency in MetS was significantly higher in individuals with low social support level compared with participants experiencing high social support. In a study conducted by Vigna et al. [47], lower social support at work was related to increased risk of MetS only among women, but not in men attending an annual routine health check-up at an occupational medicine clinic. Thus, these contradictory results may be explained by the differences in the study sample characteristics (e.g., age, gender, ethnicity, socioeconomic status) and instruments used for the evaluation of social support level.

To the best of our knowledge, no other studies assessed the combined effect of HCC and social support level on the prevalence of MetS. Results showed that there is a tendency of increased prevalence of MetS in case of a combination of higher HCC and lower social support level. Our results showed statistically significant MetS predictive effect for the highest hair cortisol tercile even after adjustment for age (2.77 fold raised odds ratio). Inclusion of other potentially confounding factors such as recreational physical activity and social support level resulted in attenuated odds ratio which was close to being statistically significant. This finding is consistent with the study of Stalder et al. [11] who found an increase in MetS prevalence with higher HCC quartile within a large occupational cohort. In contrast, cross-sectional study by Langerak et al. [15] showed that higher risk of MetS was associated with lower HCC (4.23 fold raised odds ratio in the lowest HCC tercile compared with the highest tercile). Authors explained these results by cortisol hypersensitivity which is characterized by low systemic cortisol concentration due to disruption in GR function. Other studies examined predictive value of blood serum and salivary cortisol levels for MetS. Results showed no statistically significant MetS predictive effect of cortisol measured in blood serum as a continuous variable (OR = 0.999, 95% CI (0.997, 1.001)) and distinct salivary cortisol parameters divided into terciles with odds ratio (95% CI), ranging from 0.94 (0.44, 2.01) to 1.43 (0.69, 2.96) for the lowest tercile compared with the top tercile (14,41). Together, these findings indicate methodological advantage of HCC measurement over the analysis of blood serum or salivary cortisol since only long-term changes in cortisol concentration were found to be associated with the increased MetS prevalence.

## 6 Conclusion

A significant finding in the current study is that chronically elevated cortisol concentration and lower social support level might be potential contributing factors to the development of MetS, while single point salivary or blood serum cortisol measurements reflect acute HPA axis responses which are not associated with metabolic disturbances comprising MetS.

## Abbreviations

11β-HSD2: 11β-hydroxysteroid dehydrogenase type 2
BP: blood pressure
GR: glucocorticoid receptor
HCC: hair cortisol concentration
HDL-C: high-density lipoprotein cholesterol
HPA: hypothalamic-pituitary-adrenal
MetS: metabolic syndrome
TAG: triacylglycerol
WC: waist circumference
